# Predictors of Survival in Women with High-Risk Endometrial Cancer and Comparisons of Sandwich versus Concurrent Adjuvant Chemotherapy and Radiotherapy †

**DOI:** 10.3390/ijerph17165941

**Published:** 2020-08-16

**Authors:** Hui-Hua Chen, Wan-Hua Ting, Hsu-Dong Sun, Ming-Chow Wei, Ho-Hsiung Lin, Sheng-Mou Hsiao

**Affiliations:** 1 Department of Obstetrics and Gynecology, Far Eastern Memorial Hospital, Banqiao, New Taipei 220409, Taiwan; thandaaye24@gmail.com (H.-H.C.); stellatingwh@yahoo.com (W.-H.T.); bitalvox@hotmail.com (H.-D.S.); wei@mail.femh.org.tw (M.-C.W.); hhlin@ntuh.gov.tw (H.-H.L.); 2 Department of Obstetrics and Gynecology, National Taiwan University College of Medicine and the Hospital, Taipei 100225, Taiwan; 3 Graduate School of Biotechnology and Bioengineering, Yuan Ze University, Taoyuan 320315, Taiwan

**Keywords:** endometrial neoplasms, recurrence, body mass index, chemotherapy, radiotherapy

## Abstract

*Background:* to elucidate the predictors of progression-free survival (PFS) and overall survival (OS) in high-risk endometrial cancer patients. *Methods:* the medical records of all consecutivewomen with high-risk endometrial cancer were reviewed. *Results:* among 92 high-risk endometrial cancer patients, 30 women experienced recurrence, and 21 women died. The 5-year PFS and OS probabilities were 65.3% and 75.9%, respectively. Multivariable Cox regression revealed that body mass index (hazard ratio (HR) = 1.11), paraaortic lymph node metastasis (HR = 11.11), lymphovascular space invasion (HR = 5.61), and sandwich chemoradiotherapy (HR = 0.15) were independently predictors of PFS. Body mass index (HR = 1.31), paraaortic lymph node metastasis (HR = 32.74), non-endometrioid cell type (HR = 11.31), and sandwich chemoradiotherapy (HR = 0.07) were independently predictors of OS. Among 51 women who underwent sandwich (*n* = 35) or concurrent (*n* = 16) chemoradiotherapy, the use of sandwich chemoradiotherapy were associated with better PFS (adjusted HR = 0.26, 95% CI = 0.08–0.87, *p* = 0.03) and OS (adjusted HR = 0.11, 95% CI = 0.02–0.71, *p* = 0.02) compared with concurrent chemoradiotherapy. *Conclusion:* compared with concurrent chemoradiotherapy, sandwich chemoradiotherapy was associated with better PFS and OS in high-risk endometrial cancer patients. In addition, high body mass index, paraaortic lymph node metastasis, and non-endometrioid cell type were also predictors of poor OS in high-risk endometrial cancer patients.

## 1. Introduction

The incidence of endometrial cancer (EC) is currently increasing, and most women with EC have a good prognosis. However, some women with high-risk EC have an increased risk for recurrence [1]. High-risk EC can be identified according to the presence of one of the following: (1) International Federation of Gynecology and Obstetrics (FIGO) 2009 stage I, endometrioid grade 3 cancer with deep myometrial invasion, lymphovascular space invasion, or both; (2) stage II or III disease; or (3) stage I-III disease with serous or clear cell histology [2].

There are a variety of postoperative adjuvant therapies for high-risk EC. The Gynecologic Oncology Group (GOG)-258 trial revealed that concurrent chemoradiotherapy (CRT) with additional chemotherapy was not associated with longer progression-free survival (PFS) than the chemotherapy only group [3]. However, the final results of the Postoperative Radiotherapy in Endometrial Cancer (PORTEC)-3 study revealed improved PFS and overall survival (OS) with concurrent CRT and additional chemotherapy compared with radiotherapy alone [2]. In addition to concurrent CRT with or without additional chemotherapy, early chemotherapy including sequential or sandwich CRT had been used for adjuvant therapy of high risk EC [4,5,6]. To the best of our knowledge, there was no consensus about the best adjuvant CRT for high-risk EC.

Knowledge about predictors of PFS and OS in high-risk EC patients is important for early intervention and prevention, especially for the therapeutic effects of adjuvant therapies. However, publications about predictors of PFS and OS in high-risk EC patients are scarce. Thus, the aim of this study was to elucidate the predictors of PFS and OS in high-risk EC patients, especially for the effect of different adjuvant therapies.

## 2. Materials and Methods

Medical records of all consecutive adult women with high-risk EC were reviewed. High-risk EC was defined as the presence of one of the following: (1) FIGO 2009 stage I, endometrioid grade 3 cancer with deep myometrial invasion, lymphovascular space invasion, or both; (2) stage II or III disease; or (3) stage I-III disease with serous, clear cell histology [2] or carcinosarcoma. Patients with uterine sarcoma or synchronous gynecologic malignancy were excluded. This retrospective study had been approved by the Research Ethics Review Committee of the hospital (No.109053-E).

Surgical stage was defined according to the FIGO 2009 staging system. The date of surgery, operation method, tumor grade, histological type, lymphovascular space invasion, number of lymph nodes examined, site, and number of lymph node metastases and ascites cytology were reviewed.

Sandwich CRT was defined as three cycles of chemotherapy after staging surgery followed by radiotherapy and then followed by another three cycles of chemotherapy. Concurrent CRT was defined as adjuvant radiation being given concurrently with chemotherapy with or without additional chemotherapy afterward. Radiotherapy included brachytherapy alone, external beam radiation (EBRT) alone, or combination [4].

Disease recurrence was assessed according to the appearance of abnormal radiological findings or histological proof from biopsy analyses, whichever occurred first. PFS was defined as the time interval from the date of surgery to clinically defined recurrence, disease progression, or the last follow-up. OS was calculated as the time interval from the date of surgery to the date of death from any cause or the last follow-up.

Stata version 11.0 (Stata Corp, College Station, TX, USA) was used for statistical analyses. A *p*-value less than 0.05 was considered statistically significant. Survival curves were generated using the Kaplan–Meier method, and differences in the survival curves were calculated with the log-rank test. A multivariable backward stepwise Cox proportional-hazards model was used to identify independent predictors of PFS and OS; that is, we included all variables in the univariate analysis, and then remove unimportant variables one in a time until all remaining variables with *p* <0.10 [7].

## 3. Results

**Figure 1:**
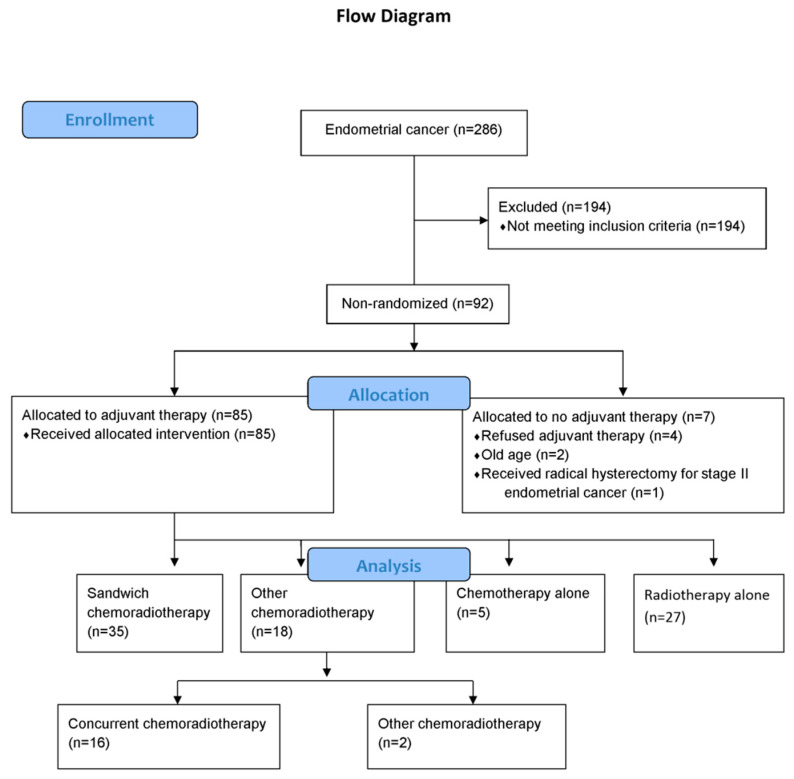
Patients with endometrial cancer.

Between January 2009 and September 2019, there were 286 EC patients, and there were 92 patients with a high-risk for EC recurrence (Figure 1). The baseline data are shown in Table 1. Seven women did not receive any adjuvant therapy. Details on the adjuvant chemotherapy and radiotherapy of the other 85 women are listed in Table 2. The causes of the seven patients who did not received adjuvant therapy include patients’ refusal (*n* = 4), old age (*n* = 2), and radical hysterectomy for her stage II EC (*n* = 1) (Figure 1).

**Figure 2:**
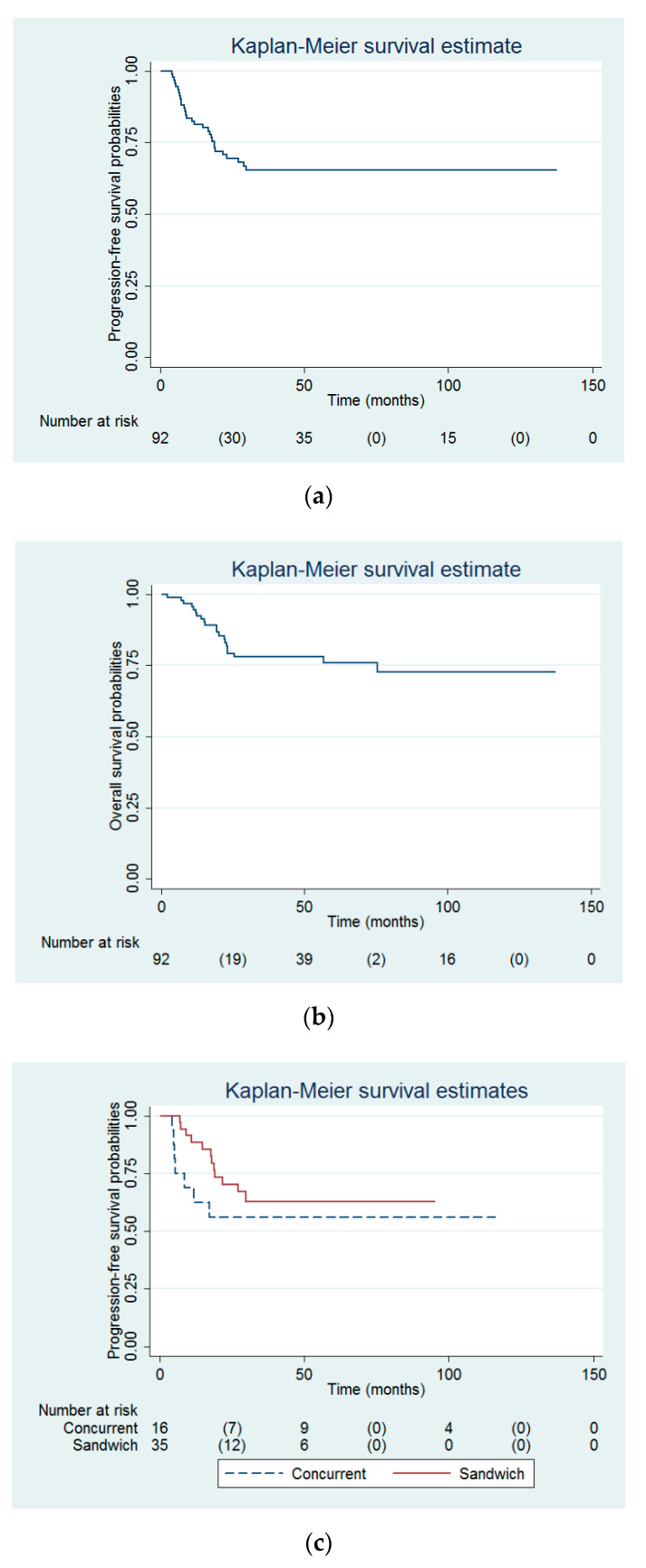

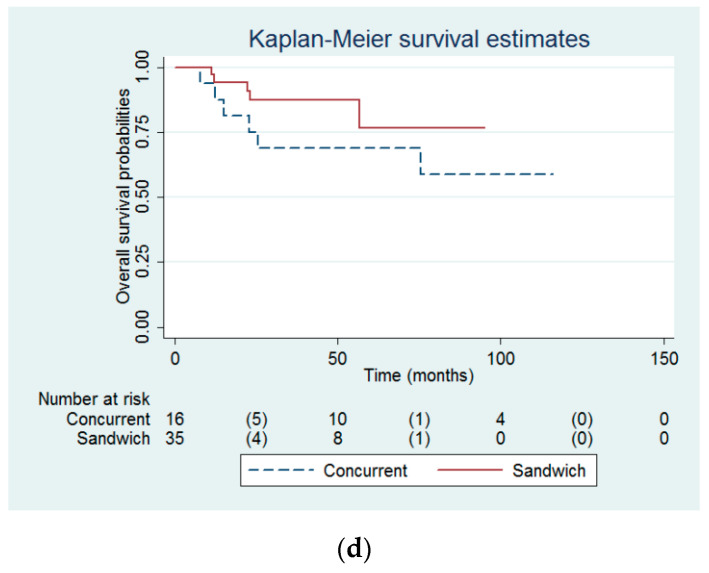
Probabilities of (**a**) progression-free survival and (**b**) overall survival of whole study population. Probabilities of (**c**) progression-free survival and (**d**) overall survival between the sandwich and concurrent chemoradiotherapy groups.

There were 30 women who experienced recurrence and 21 women who died. The 3 year and 5-year PFS probability were 65.3% (95% confidence interval (CI) = 54.1–74.4%) and 65.3% (95% CI = 54.1–74.4%, Figure 2a), respectively. The 3-year and 5-year OS probability were 77.9% (95% CI = 67.6–85.4%) and 75.9% (95% CI = 64.8–83.9%, Figure 2b), respectively.

Four patients received surgeries for their recurrence, including craniotomy, debulking surgery, video-assisted thoracoscopic surgery resection of lingula lobe, and tumor excision in spine. Three patients remained alive during follow-up.

Multivariable Cox proportional-hazards modeling revealed that body mass index (hazard ratio (HR) = 1.11), paraaortic lymph node metastasis (HR = 11.11), lymphovascular space invasion (HR = 5.61), and sandwich CRT (HR = 0.15) were the independent predictors for PFS (Table 3).

Multivariable Cox proportional-hazards modeling showed that body mass index (HR = 1.31), paraaortic lymph node metastasis (HR = 32.74), non-endometrioid cell type (HR = 11.31) and sandwich CRT (HR = 0.07) were the independent predictors for OS (Table 4).

There were 53 women who received combined CRT for high-risk EC, including sandwich CRT (*n* = 35) and other combined CRT (*n* = 18). Among those receiving other combined CRT, 16 of 18 women received concurrent CRT, and 4 of the above 16 concurrent CRT women received additional chemotherapy afterward (Table 2).

Among the 51 women who underwent sandwich (*n* = 35) or concurrent (*n* = 16) CRT. There were no between-group differences in the total doses of EBRT (51.0 ± 6.2 Gy vs. 50 ± 4.5 Gy, *p* = 0.67) and brachytherapy (17.1 ± 5.7 Gy high dose rate vs. 17.4 ± 5.7 Gy high dose rate, *p* = 0.59) between the concurrent and sandwich CRT groups. In addition, there were no between-group differences in the patient numbers with interrupted therapy of chemotherapy (5 cases vs. 6 cases, *p* = 0.29) and chemotherapy cycles (5.9 ± 1.7 cycles vs. 5.7 ± 0.9 cycles, *p* = 0.89) between the concurrent and sandwich CRT groups. Multivariable Cox proportional-hazards modeling revealed that pelvic lymph node number (HR = 0.92), paraaortic lymph node metastasis (HR = 7.64), and sandwich CRT (HR = 0.26, Figure 2c) were the independent predictors for PFS (Table 5). In addition, multivariable Cox proportional-hazards modeling showed body mass index (HR = 1.24), paraaortic lymph node metastasis (HR = 64.06), and sandwich CRT (HR = 0.11, Figure 2d) were the independent predictors for OS (Table 6).

## 4. Discussion

In this study, the use of sandwich CRT was associated with better PFS (adjusted HR = 0.26, Table 5, Figure 2c) and OS (adjusted HR = 0.11, Table 6, Figure 2d), compared with concurrent CRT. To our knowledge, there was no article mentioned about the comparison between sandwich and concurrent CRTs. Similarly, Onal et al. reported a superiority of sandwich CRT in PFS and OS, compared with sequential CRT or radiotherapy only [8,9]. The underlying mechanism explaining the finding about the superiority of sandwich CRT is unknown. However, the findings of this current study echo the hypothesis that combined CRT might be weakened by giving sensitizer (i.e., concurrent CRT) rather than full-dose chemotherapy (i.e., sandwich CRT in this study) upfront [4]. Similarly, Goodman et al. also reported that multi-agent chemotherapy followed by radiotherapy (or potentially employing a sandwich approach) may optimize control of both locoregional and distant disease, ultimately leading to longer OS, compared with the treatment with radiotherapy followed by chemotherapy for patients with advanced EC [10]. Elemam et al. found that adjuvant chemotherapy given before radiotherapy may lessen the effect of high-risk features on disease-free survival and OS [11]. Nonetheless, Boothe et al. reported that there was no difference of OS between early chemotherapy (e.g., sequential or sandwich CRT) and late chemotherapy (e.g., concurrent CRT) [4].

In this study, paraaortic lymph node metastasis was a predictor for PFS (HR = 11.11, Table 3) and OS (HR = 32.74, Table 4). Similarly, Young et al. reported that positive lymph node metastasis was associated with worse PFS (HR = 3.37, *p* = 0.03) and OS (HR = 2.96, *p* = 0.04) in FIGO 1988 stage III EC patients [12]. Onal et al. also found that paraaortic lymph node metastasis was a predictor for worse OS (HR = 2.01, 95% CI = 1.15–3.53, *p* = 0.02) compared with pelvic lymph node metastasis [9]. Thus, patients with paraaortic lymph node metastasis might be treated with a more intensive therapy to prevent EC recurrence. The negative role of paraaortic lymph node metastasis may highlight the importance of paraaortic lymph node dissection, even with minimally invasive techniques and in obese patients [13].

Non-endometrioid cell type included serous cell carcinoma, clear cell carcinoma, and carcinosarcoma. Women with non-endometrioid cell type have a poor prognosis compared with endometrioid cell type. Thus, it seems reasonable that non-endometrioid cell type was a predictor of poor OS (HR = 11.31, Table 4) in our study.

In this current study, the use of sandwich CRT were statistically non-significant in the univariate analysis of PFS (HR = 0.64, *p* =0.35, Table 5) and OS (HR = 0.48, *p* =0.24, Table 6), but significant in the multivariable analysis of PFS (adjusted HR = 0.26, *p* =0.03, Table 5) and OS (adjusted HR = 0.11, *p* =0.02, Table 6). Some scenarios, such as the influence of missing data, the effect of an unbalanced sample size, the presence of interactions and an extremely large within-group variation, may result in some variables being non-significant in the univariate analysis but significant in the multivariable analysis [14,15].

In this current study, robotic or laparoscopic approach was not a predictor of PFS or OS (Table 3; Table 4). Similarly, the GOG-LAP2 study reported that the potential of laparoscopic treatment for increased risk of cancer recurrence was small [16]. In addition, minimally invasive surgery was reported to have a predominant role in EC patients with important morbidity and in the elderly population [17].

In this study, four patients received surgeries for their recurrence. In general, criteria for surgery of EC recurrence include good performance, no other distant metastasis, and resection of macroscopic residual disease likely achievable [18]. It was reported that complete cytoreductive surgery was associated with prolonged post-recurrence survival compared to patients left with any gross residual disease [19].

Adjuvant chemotherapy seems to be important for locally advanced EC. A Cochrane review reported that chemotherapy increases survival time by approximately 25% related to radiotherapy in stage III and IV EC [20]. In the sandwich and concurrent CRT groups, 72.5% (37/51) patients received ≥6 cycles of chemotherapy (Table 2). Similarly, the patients received six cycles of chemotherapy in the GOG-258 trial [3]. In addition, the patients received two cycles of cisplatin and four cycles of carboplatin/paclitaxel in the PORTEC-3 trial [2]. Kim et al. also reported that ≥6 cycles of chemotherapy may be more beneficial than 3–5 cycles of chemotherapy for high-risk endometrioid EC patients [21]. Nonetheless, Mayam et al. reported that four cycles of chemotherapy may be suitable for high-risk EC patients due to low incidence of hematologic toxicities without impairing survival compared with six cycles [22]. Thus, the optimal cycles of adjuvant chemotherapy for high risk EC seems to be undetermined.

In the sandwich CRT group, 94.3% (33/35) patients received platinum/paclitaxel or platinum/doxorubicin for adjuvant chemotherapy (Table 2). The Japanese Gynecologic Oncology Group reported that the efficacies were similar between the platinum/paclitaxel and cisplatin/doxorubicin regimens [23]. Thus, the finding about the superiority of the sandwich CRT might be extrapolated to the sandwich CRT patients who received platinum/paclitaxel or platinum/doxorubicin as their sole adjuvant chemotherapy regimen.

EBRT with or without vaginal brachytherapy has been used as the main radiotherapy regimen for high risk EC [2,3]. In the PORTEC-3 trial, 48.6 Gy was recommended for EBRT; and vaginal brachytherapy with equivalent to 14 Gy was given for case of cervical involvement [2]. In the GOG-258 trial, 45 Gy was recommended for EBRT, and vaginal brachytherapy with 12–18 Gy high dose rate or 20–35 Gy low dose rate was given for cervical, low segment or vagina1 involvement, or lymphovascular space invasion [3]. In the current study, the EBRT (i.e., 49.5 ± 4.6 Gy) and vaginal brachytherapy doses (i.e., 19.7 ± 7.0 Gy high dose rate) seems to be similar to that of the PORTEC-3 and GOG-258 trials. Nonetheless, it is worth mentioning that the stage I-II serous, clear cell and high-grade endometrioid EC patients may not benefit from the addition of brachytherapy to EBRT [24]; and vaginal brachytherapy alone may be reasonable for stage II endometrioid EC patients with adequate lymph node dissection and low-grade tumors [25].

Limitations of this study include the retrospective nature of the study, heterogeneous adjuvant therapy, limited sample size, and non-randomization. However, detailed person-time data might increase the reliability of this study. In addition, the discrepancy in the percentage of multi-agent chemotherapy (i.e., 100%, 35/35, in the sandwich CRT group vs. 31.3%, 5/16, in the concurrent CRT group, *p* <0.001) may bias the result. Future randomized controlled trials are suggested to confirm the finding of this study.

## 5. Conclusions 

Compared with concurrent CRT, sandwich CRT was associated with better PFS and OS in high-risk EC patients. In addition, high body mass index, paraaortic lymph node metastasis, and non-endometrioid cell type were also predictors of poor OS in high-risk EC patients.

## Figures and Tables

**Table 1:** Baseline data of high-risk endometrial cancer patients who underwent adjuvant therapy (*n* = 92).

Variables	Values
Age (years)	58.6 ± 10.5
Parity (*n*)	2.0 ± 1.6
Body mass index (kg/m^2^)	25.6 ± 4.1
Diabetes	17 (18)
CA125 (U/mL)	188.4 ± 887.0
ECOG	
0	52 (57)
1	37 (40)
2	2 (2)
3	1 (1)
FIGO stage	
IA	17 (18)
IB	10 (11)
II	17 (18)
IIIA	10 (11)
IIIC1	26 (28)
IIIC2	12 (13)
Robotic/laparoscopic approach	17 (18)
Paraaortic lymph node sampling/dissection	62 (67)
Total pelvic lymph node number (*n*)	16.7 ± 8.3
Total paraaortic lymph node number (*n*)	4.9 ± 4.3
Presence of pelvic lymph node metastasis	32 (35)
Presence of paraaortic lymph node metastasis	12 (13)
Presence of LVSI	62 (67)
Presence of deep myometrial invasion	49 (53)
Tumor size (cm)	4.8 ± 2.5
Presence of malignant cell in washing cytology	6 (7)
Non-endometrioid cell type	36 (39)
Adjuvant therapy	
Sandwich chemoradiotherapy	35 (38)
Other chemoradiotherapy	18 (20)
Radiotherapy only	27 (29)
Chemotherapy only	5 (5)
No adjuvant therapy	7 (8)
Follow-up interval (months)	52.9 ± 37.5
Recurrence	30 (33)
Death	21 (23)

Values are expressed as mean ± standard deviation or number (percentage). BMI = body mass index, CA125 = cancer antigen 125, CI = confidence interval, ECOG = Eastern Cooperative Oncology Group, FIGO = The International Federation of Gynecology and Obstetrics, LVSI = lymphovascular space invasion.

**Table 2:** Chemotherapy and radiotherapy regimens, and subsequent therapies for recurrence for women with high-risk endometrial cancer (*n* = 92).

Variables	Regimens	Sandwich CRT (*n* = 35)	Other CRT (*n* = 18)	CT Only (*n* = 5)	RT Only (*n* = 27)	No Adjuvant Treatment (*n* = 7)	^†^ *p*
Age (years)	-	57.3 ± 10.6	57.4 ± 10.6	57.8 ± 6.4	58.7 ± 9.0	68.4 ± 15.2	0.14
Stage II	-	4 (11)	2 (11)	1 (0)	9 (33)	1 (14)	<0.0001
Stage III	-	28 (80)	14 (74)	2 (40)	4 (15)	0 (0)	
Endometrioid	-	20 (57)	14 (79)	4 (80)	20 (74)	1 (14)	0.054
Clear cell	-	8 (23)	3 (16)	1 (20)	1 (4)	1 (14)	
Serous	-	2 (6)	0 (0)	0 (0)	2 (7)	1 (14)	
Carcinosarcoma	-	5 (14)	1 (5)	0 (0)	4 (15)	4 (57)	
CT regimen	Platinum/paclitaxel	26 (74)	2 (11)	3 (60)	-	-	
	Platinum/doxorubicin	7 (20)	2 (11)	2 (40)	-	-	
	Ifosfamide/paclitaxel	2 (6)	1 (5)	-	-	-	
	Platinum	-	11 (58)	-	-	-	
	Platinum/doxorubicin/palitaxel	-	1 (5)	-	-	-	
	Platinum/cyclophosphamide	-	1 (5)	-	-	-	
RT regimen	EBRT + Brachytherapy	12 (34)	8 (42)	-	8 (30)	-	
	EBRT	21 (60)	10 (58)	-	13 (48)	-	
	Brachytherapy	2 (6)	-	-	6 (22)	-	
Duration of adjuvant treatment (months)		6.4 ± 1.6	2.6 ± 1.8	3.3 ± 1.5	0.7 ± 2.4	0 ± 0	<0.001
Recurrence		12 (34)	8 (42)	3 (60)	6 (22)	1 (14)	0.26
Local recurrence		5 (14)	4 (21)	3 (60)	3 (11)	0 (0)	0.051
Distant metastasis		8 (23)	4 (21)	1 (20)	4 (15)	1 (14)	0.93
Death		5 (14)	8 (42)	2 (40)	4 (15)	2 (29)	0.09
Subsequent RT for recurrence		3 (9)	7 (39)	1 (20)	3 (11)	0 (0)	0.08
Subsequent CT for recurrence		4 (11)	4 (22)	2 (40)	3 (11)	1 (14)	0.58
Subsequent surgery for recurrence		2 (6)	1 (6)	0 (0)	1 (4)	0 (0)	1.00
Subsequent HT for recurrence		3 (9)	1 (6)	0 (0)	2 (7)	0 (0)	0.77

Values are expressed as mean ± standard deviation, median (25–75 interquartile range) or number (percentage). CRT = chemoradiotherapy, CT = chemotherapy, EBRT = external beam radiotherapy, HT = hormone therapy, RT = radiotherapy. ^†^ Analysis of variance or Fisher’s exact test.

**Table 3:** Univariate and multivariable Cox proportional-hazards model for predicting progression free survival after adjuvant therapy for women with high risk endometrial cancer (*n* = 92).

Variable		Univariate			Multivariable	
	Hazard Ratio	95% CI	^†^ *p*	Hazard Ratio	95% CI	^‡^ *p*
Age (years)	1.02	0.99–1.06	0.13	-	-	-
Parity (*n*)	1.08	0.87–1.35	0.48	0.70	0.49–1.00	0.053
Body mass index (kg/m^2^)	1.11	1.01–1.21	0.02	1.11	1.01–1.24	0.03
Diabetes (yes vs. no)	1.50	0.64–3.49	0.35	-	-	-
CA125 (U/mL)	1.00	1.00–1.00	0.31	-	-	-
ECOG score (*n*)	1.64	0.97–2.79	0.06	-	-	-
FIGO stage (IA = 1, IB = 2, II = 3, IIIA = 4, IIIC1 = 5, IIIC2 = 6)	1.50	1.15–1.95	0.003	-	-	-
Robotic/laparoscopic approach (yes vs. no)	1.06	0.40–2.76	0.91	-	-	-
Paraaortic lymph node sampling/dissection (yes vs. no)	1.23	0.56–2.69	0.60	-	-	-
Total pelvic lymph node number (*n*)	0.97	0.93–1.02	0.23	-	-	-
Total paraaortic lymph node number (*n*)	1.02	0.92–1.13	0.69	0.86	0.74–1.00	0.051
Presence of pelvic lymph node metastasis	1.82	0.89–3.73	0.10	-	-	-
Presence of paraaortic lymph node metastasis	4.10	1.71–9.85	0.002	11.11	3.27–37.80	<0.001
Presence of LVSI	4.53	1.07–19.12	0.04	5.61	1.05–29.89	0.04
Presence of deep myometrial invasion	1.29	0.62–2.67	0.50	-	-	-
Tumor size (mm)	1.02	1.01–1.03	0.002	-	-	-
Presence of malignant cell in washing cytology	1.99	0.60–6.64	0.26	-	-	-
Non-endometrioid cell type (yes vs. no)	0.68	0.31–1.49	0.34	-	-	-
Cell grade (*n*)	0.85	0.50–1.42	0.53	-	-	-
Brachytherapy (yes vs. no)	0.69	0.32–1.51	0.36	-	-	-
Sandwich chemoradiotherapy (yes vs. no)	0.97	0.47–2.02	0.94	0.15	0.05–0.48	0.001

The abbreviations are the same as in Table 1. ^†^ Cox proportional-hazards model. ^‡^ Multivariable backward stepwise Cox proportional-hazards modeling was performed by using all variables in the univariate analysis until all remaining variable(s) with *p* < 0.10.

**Table 4:** Univariate and multivariable Cox proportional-hazards model for predicting overall survival after adjuvant therapy for women with high-risk endometrial cancer (*n* = 92).

Variable		Univariate			Multivariable	
	Hazard Ratio	95% CI	^†^ *p*	Hazard Ratio	95% CI	^‡^ *p*
Age (years)	1.02	0.98–1.06	0.34	-	-	-
Parity (*n*)	1.01	0.77–1.33	0.93	0.68	0.45–1.04	0.08
Body mass index (kg/m^2^)	1.13	1.02–1.25	0.02	1.31	1.11–1.52	0.001
Diabetes (yes vs. no)	1.60	0.59–4.38	0.36	-	-	-
CA125 (U/mL)	1.00	1.00–1.00	0.68	-	-	-
ECOG score (*n*)	1.64	0.89–3.01	0.11	-	-	-
FIGO stage (IA = 1, IB = 2, II = 3, IIIA = 4, IIIC1 = 5, IIIC2 = 6)	1.06	0.82–1.39	0.65	-	-	-
Robotic/laparoscopic approach	1.54	0.56–4.22	0.40	-	-	-
Paraaortic lymph node sampling/dissection (yes vs. no)	1.43	0.55–3.68	0.46	-	-	-
Total pelvic lymph node number (*n*)	1.02	0.97–1.07	0.54	-	-	-
Total paraaortic lymph node number (*n*)	1.07	0.98–1.16	0.16	-	-	-
Presence of pelvic lymph node metastasis	0.84	0.34–2.08	0.70	-	-	-
Presence of paraaortic lymph node metastasis	2.56	0.87–7.54	0.09	32.74	5.09–210.61	<0.001
Presence of LVSI	1.27	0.42–3.83	0.67	-	-	-
Presence of deep myometrial invasion	0.80	0.34–1.88	0.61	-	-	-
Tumor size (mm)	1.01	1.00–1.03	0.17	-	-	-
Presence of malignant cell in washing cytology	1.52	0.35–6.58	0.58	-	-	-
Non-endometrioid cell type (yes vs. no)	1.14	0.48–2.71	0.77	11.31	2.40–53.31	0.002
Cell grade (*n*)	1.12	0.57–2.17	0.75	-	-	-
Brachytherapy (yes vs. no)	1.49	0.97–2.30	0.07	-	-	-
Sandwich chemoradiotherapy (yes vs. no)	0.52	0.19–1.44	0.21	0.07	0.01–0.32	0.001

The abbreviations are the same as in Table 1. ^†^ Cox proportional-hazards model. ^‡^ Multivariable backward stepwise Cox proportional-hazards modeling was performed by using all variables in the univariate analysis until all remaining variable(s) with *p* < 0.10.

**Table 5:** Univariate and multivariable Cox proportional-hazards model for predicting progression-free survival after adjuvant therapy with sandwich (*n* = 35) or concurrent (*n* = 16) chemoradiotherapy.

Variable		Univariate			Multivariable	
	Hazard Ratio	95% CI	^†^ *p*	Hazard Ratio	95% CI	^‡^ *p*
Age (years)	1.07	1.02–1.12	0.006	-	-	-
Parity (*n*)	1.21	0.90–1.62	0.20	-	-	-
Body mass index (kg/m^2^)	1.08	0.97–1.20	0.15	-	-	-
Diabetes (yes vs. no)	0.87	0.20–3.76	0.85	-	-	-
CA125 (U/mL)	1.001	0.998–1.004	0.55	-	-	-
ECOG score (*n*)	1.58	0.81–3.09	0.18	-	-	-
FIGO stage (IA = 1, IB = 2, II = 3, IIIA = 4, IIIC1 = 5, IIIC2 = 6)	2.85	1.49–5.44	0.002			
Robotic/laparoscopic approach (yes vs. no)	1.23	0.36–4.24	0.74	-	-	-
Paraaortic lymph node sampling/dissection (yes vs. no)	1.34	0.44–4.03	0.61	-	-	-
Total pelvic lymph node number (*n*)	0.92	0.86–0.98	0.01	0.92	0.85–1.00	0.04
Total paraaortic lymph node number (*n*)	1.02	0.92–1.14	0.65			
Presence of pelvic lymph node metastasis	1.67	0.63–4.41	0.30	-	-	-
Presence of paraaortic lymph node metastasis	5.08	1.76–14.61	0.003	7.64	2.28–25.67	0.001
Presence of LVSI	2.11 × 10^15^	0–infinity	1.00	-	-	-
Presence of deep myometrial invasion	1.51	0.54–4.18	0.43	-	-	-
Tumor size (mm)	1.02	1.00–1.04	0.01	-	-	-
Presence of malignant cell in washing cytology	1.90	0.55–6.65	0.31	-	-	-
Non-endometrioid cell type (yes vs. no)	0.69	0.26–1.82	0.46	-	-	-
Cell grade (*n*)	0.76	0.39–1.50	0.44	-	-	-
Brachytherapy (yes vs. no)	0.83	0.33–2.12	0.70	-	-	-
Sandwich chemoradiotherapy (yes vs. no)	0.64	0.25–1.63	0.35	0.26	0.08–0.87	0.03

The abbreviations are the same as in Table 1. ^†^ Cox proportional-hazards model. ^‡^ Multivariable backward stepwise Cox proportional-hazards modeling was performed by using all variables in the univariate analysis until all remaining variable(s) with *p* < 0.10.

**Table 6:** Univariate and multivariable Cox proportional-hazards model for predicting overall survival after adjuvant therapy with sandwich (*n* = 35) or concurrent (*n* = 16) chemoradiotherapy.

Variable		Univariate			Multivariable	
	Hazard Ratio	95% CI	^†^ *p*	Hazard Ratio	95% CI	^‡^ *p*
Age (years)	1.05	0.99–1.12	0.10	-	-	-
Parity (*n*)	1.03	0.70–1.52	0.86	0.58	0.30–1.11	0.097
Body mass index (kg/m^2^)	1.11	0.96–1.28	0.15	1.24	1.02–1.52	0.03
Diabetes	1.94	0.41–9.16	0.40	-	-	-
CA125 (U/mL)	1.001	0.997–1.005	0.56	-	-	-
ECOG score (*n*)	2.07	0.94–4.58	0.07	-	-	-
FIGO stage (IA = 1, IB = 2, II = 3, IIIA = 4, IIIC1 = 5, IIIC2 = 6)	1.97	0.97–4.01	0.06	-	-	-
Robotic/laparoscopic approach (yes vs. no)	0.54	0.07–4.22	0.56	-	-	-
Paraaortic lymph node sampling/dissection (yes vs. no)	0.91	0.24–3.45	0.90	-	-	-
Total pelvic lymph node number (*n*)	0.98	0.92–1.05	0.57	-	-	-
Total paraaortic lymph node number (*n*)	1.09	0.99–1.20	0.07	-	-	-
Presence of pelvic lymph node metastasis	0.61	0.19–2.02	0.42	-	-	-
Presence of paraaortic lymph node metastasis	5.11	1.20–21.70	0.07	64.06	3.09–1329.06	0.007
Presence of LVSI	7.55 × 10^14^	0–infinity	1.00	-	-	-
Presence of deep myometrial invasion	2.58	0.56–12.00	0.23	-	-	-
Tumor size (mm)	1.02	1.00–1.05	0.02	-	-	-
Presence of malignant cell in washing cytology	1.57	0.33–7.49	0.57	-	-	-
Non-endometrioid cell type (yes vs. no)	0.88	0.26–3.00	0.84	10.99	0.92–131.58	0.06
Cell grade (*n*)	0.68	0.27–1.70	0.41	-	-	-
Brachytherapy (yes vs. no)	1.96	0.59–6.48	0.27	-	-	-
Sandwich chemoradiotherapy (yes vs. no)	0.48	0.14–1.64	0.24	0.11	0.02–0.71	0.02

The abbreviations are the same as in Table 1. ^†^ Cox proportional-hazards model. ^‡^ Multivariable backward stepwise Cox proportional-hazards modeling was performed by using all variables in the univariate analysis until all remaining variable(s) with *p* < 0.10.
